# Characterization of early-stage lesions and investigation on the role of mucosal trauma in hemorrhagic bowel syndrome in cattle

**DOI:** 10.1080/01652176.2024.2360422

**Published:** 2024-06-04

**Authors:** Bert De Jonge, Bart Pardon, Jozefien Callens, Koen Chiers

**Affiliations:** a Department of Pathobiology, Pharmacology and Zoological Medicine, Ghent University, Merelbeke, Belgium; b Department of Internal Medicine, Reproduction and Population Medicine, Ghent University, Merelbeke, Belgium; c Animal Health Service-Flanders (DGZ Vlaanderen), Torhout, Belgium

**Keywords:** Jejunal hemorrhage syndrome, dairy, hematoma, abrasion, intestine, pathology

## Abstract

Hemorrhagic bowel syndrome (HBS) is characterized by a dissecting intramucosal hematoma at the small bowel, causing obstruction and severe hemorrhage in dairy cattle. Recent investigation revealed the presence of early-stage lesions in cows affected by HBS. These are presumed to be the initial stage of the hematoma, as both share unique dissection of the lamina muscularis mucosae (LMM) as histological hallmark. Early-stage lesions of HBS have not been characterized in greater detail, and neither has the hypothesis of mucosal abrasion as etiology been explored. Therefore, the first objective of the present study was to characterize the morphology of early-stage lesions, by gross examination, histochemistry, immunohistochemistry and transmission electron microscopy. The second objective was to determine the effect of mucosal abrasion to the small intestine in an *ex vivo* model. A total of 86 early-stage lesions from 10 cows with HBS were characterized. No underlying alterations at the LMM were evident which could explain their occurrence. However, degeneration at the ultrastructural level of the LMM smooth muscle cells was present in 3 of 4 lesions, it is however unclear whether this is primary or secondary. Bacteriological examination did not reveal any association with a specific bacterium. Experimental-induced and early-stage lesions were gross and histologically evaluated and scored in three cows with HBS and seven controls. Experimentally induced lesions in both affected cows and controls, were histologically very similar to the naturally occurring early-stage lesions. Altogether, the results are suggestive for mucosal trauma to play a role in the pathogenesis of HBS.

## 1. Introduction

Hemorrhagic Bowel Syndrome (HBS) or Jejunal hemorrhage syndrome (JHS), is a sporadic, acute intestinal disease of mainly adult dairy cattle (Elhanafy et al. 2013), and in two studies brown Swiss cattle were overrepresented (Peek et al. 2009; Braun et al. 2010). HBS has a rapid clinical course with animals showing signs of intestinal obstruction and shock, with death often within 12–48h (Peek et al. 2018). Even with surgical intervention, the prognosis is reserved and recurrence is common (Peek et al. 2009). HBS is characterized by a solitary and dissecting intramucosal hematoma at the small bowel (De Jonge et al. 2023). This hematoma causes intestinal obstruction, and severe intraluminal hemorrhage. It is assumed that the hematoma develops from small mucosal erosions-lacerations, or so called early-stage lesions, through dissecting hemorrhage within the lamina muscularis mucosae (LMM), a thin bilayer of smooth muscle tissue within the mucosa (De Jonge et al. 2023). Etiology and pathogenesis are currently unknown. *Clostridium perfringens* type A has drawn much attention as possible etiologic agent (Kirkpatrick et al. 2001; Abutarbush and Radostits 2005; Ceci et al. 2006), but more recent work does not support this hypothesis (Adaska et al. 2014; De Jonge et al. 2023). In these previous studies, bacterial culture was often performed at the hematoma or small intestine, but not at these early-stage lesions. Potentially, a causative pathogen can only be retrieved in the early-stage lesion.

These early-stage lesions are grossly characterized by erosion or laceration of the mucosa with variable hemorrhage. Histologically, the hematoma and early-stage lesions both share the feature of detachment of the mucosa through splitting of the LMM (De Jonge et al. 2023). There are however no primary histological lesions which might explain the development of these early-stage lesions. Also grossly, some early-stage lesions have a laceration-like appearance. Based on both findings, it was hypothesized that mucosal trauma might play a role in the pathogenesis of these lesions (De Jonge et al. 2023). Furthermore, because Brown Swiss or related breeds, are overrepresented in several studies (Peek et al. 2009; Braun et al. 2010), and re-occurrence within months of an HBS-episode in a recovered animal is often observed (Peek et al. 2009; Braun et al. 2010), an underlying condition predisposing animals to HBS could be suspected. Combined with the consistent splitting of the LMM as a primary lesion, this could point at some kind of alteration of the smooth muscle tissue of the LMM. An abnormal LMM could be predisposed to rupture and eventually splitting because of mechanical stress created by peristalsis and digesta passage. To date, no studies have investigated this hypothesis yet.

Therefore, the aim of this study was to gain further insight into the pathogenesis of HBS. Firstly, it was evaluated if any structural alterations are present at the LMM of early-stage lesions derived from 10 cows, through histological, immunohistological and ultrastructural examination. Additionally, gross pathology and bacterial culture results are described. Secondly, we developed an *ex vivo* model to produce mucosal abrasion, used to test if lesions similar to HBS early-stage lesions can be produced. Also, small intestine of cows with HBS and controls (dairy and beef cattle) were compared with this model, to examine if any increased mucosal fragility is present, possibly explaining the individual tendency of animals to develop HBS.

## 2. Materials and methods

### 2.1. Characterization of HBS early-stage lesions

#### 2.1.1. Animals and necropsy

Ten cows with HBS were enrolled in this study. Four animals were euthanized or died at the dairy farm and were immediately transported to the university necropsy facility. Six cows with clinical suspicion of HBS were transported to the Clinic of Large Animal Internal Medicine of Ghent University. Three animals were further clinically assessed and euthanatized if HBS was very likely and the owner choose not to proceed treatment. Two animals were immediately euthanized upon arrival because of clinical deterioration and recumbency. One animal was surgically treated, and died the following day.

All cows were necropsied within 1 h after death. The diagnosis of HBS was made by identifying the typical intestinal hematoma. All organs were grossly examined. The complete small intestine was opened and evaluated for mucosal erosions or lacerations (HBS early-stage lesions), as previously described (De Jonge et al. 2023). The number of lesions and their location were recorded.

#### 2.1.2. Histology and immunohistochemistry

From all cases (*n* = 10), 86 lesions showing mucosal erosion were completely sampled and fixed in 4% buffered formaldehyde for 24–72 h. Subsequently, all specimens were routinely processed, cut at 5-µm-thick sections and stained with hematoxylin and eosin (HE). From each cow, one early-stage lesion showing hemorrhage, and one sample of grossly normal small intestine, were further examined. These samples were stained with periodic acid-Schiff (PAS) and Gomori’s reticulin stain. In addition, samples were immunolabeled for laminin, desmin and alpha-smooth muscle actin. In brief, antigen retrieval was obtained by immersion of FFPE samples in citrate-buffered (0.01 M, pH 6). For laminin an enzymatic antigen retrieval (proteinase K, Agilent) was performed. After blocking of endogenous peroxidase, slides were incubated with polyclonal rabbit antibody directed against laminin (1/200, Z0097, Agilent, Santa Clara, California, US); monoclonal mouse antibody against desmin (1/200, M0760, Agilent, Santa Clara, California, US) or monoclonal mouse antibody against smooth muscle actin (1/200, M085101, Agilent, Santa Clara, California, US). This was done at room temperature for 30 min with an antibody diluent solution with background-reducing components (S302283-2, Agilent). After incubation with polymer-based secondary anti-rabbit antibody (K400311, Agilent) and polymer-based secondary anti-mouse antibody (K400111, Agilent) at room temperature for 30 min, visualization was performed in a 3.3-diaminobenzidine solution (K346811, Agilent) at room temperature for 5 min. Visualization was performed using an EnVision + System-HRP (Dako). Four sections of small intestine of dairy cows which died from non-intestinal diseases were used as controls.

#### 2.1.3. Transmission electron microscopy

Four early-stage lesions and one hematoma, obtained from three different animals were sampled for transmission electron microscopy (TEM). The sample location was for both hematoma and early-stage lesion, at the transition with normal mucosa. Three intestinal samples from non-affected dairy cows derived from the slaughterhouse were used as controls with 2/3 being abrasional lesions created with the *ex vivo* model (see below). All samples were initially fixated in 10% buffered formalin within 4 h post-mortem. After formalin fixation, samples were transferred to Karnovsky’s fixative before 4 washes with 0.1 M sodium cacodylate buffer and post-fixation in 1% osmium tetroxide. After fixation, tissues were washed with double-distilled water followed by dehydration in a graded series of alcohol before infiltration and embedding in Spurr’s epoxy resin. Semi-thin sections were cut at 1 μm and stained with toluidine blue to select areas for ultrathin sectioning. Ultrathin sections (90 nm) were collected on Formvar-coated copper grids. Sections were stained with uranyl acetate and lead citrate and examined with a JEM − 1400 Plus transmission electron microscope (JEOL, Benelux).

#### 2.1.4. Bacteriology

In order to identify a possible etiological bacterium, mucosal swabs were taken from 15 early-stage lesions with hemorrhage. These lesions were derived from six animals with HBS, with the number of sampled lesions ranging from one to five per animal. Swabs were stored at 4 °C for less than 12h, and inoculated within 12h after sampling on two Columbia agar plates supplemented with 5% sheep blood (Oxoïd, Hampshire, UK). Plates were incubated for 24 h at 37 °C in either aerobic or anaerobic conditions. Furthermore, a selective chromogenic culture medium for *C. perfringens* was used (CHROMagar C perfringens, CHCP), allowing a better qualitative and semiquantitative estimation (Hustá et al. 2020). After incubation of non-selective plates, 4 to 7 colonies from different morphologies were further purified and identified with an Autoflex III smartbeam MALDI-TOF mass spectrometry, using FlexControl and MBT Compass software (Bruker Daltonics, Bremen, Germany) as described before (Hustá et al. 2020). A semiquantitative estimation of the abundance of *C. perfrigens* growth at the CHCP plate was made by scoring according to 0 for no growth, + and ++ for respectively <33% and >33% of the streaked area containing *C. perfringens* colonies.

### 2.2. Ex vivo intestinal mucosal abrasion model

#### 2.2.1. Description of the model

**Figure 1.**
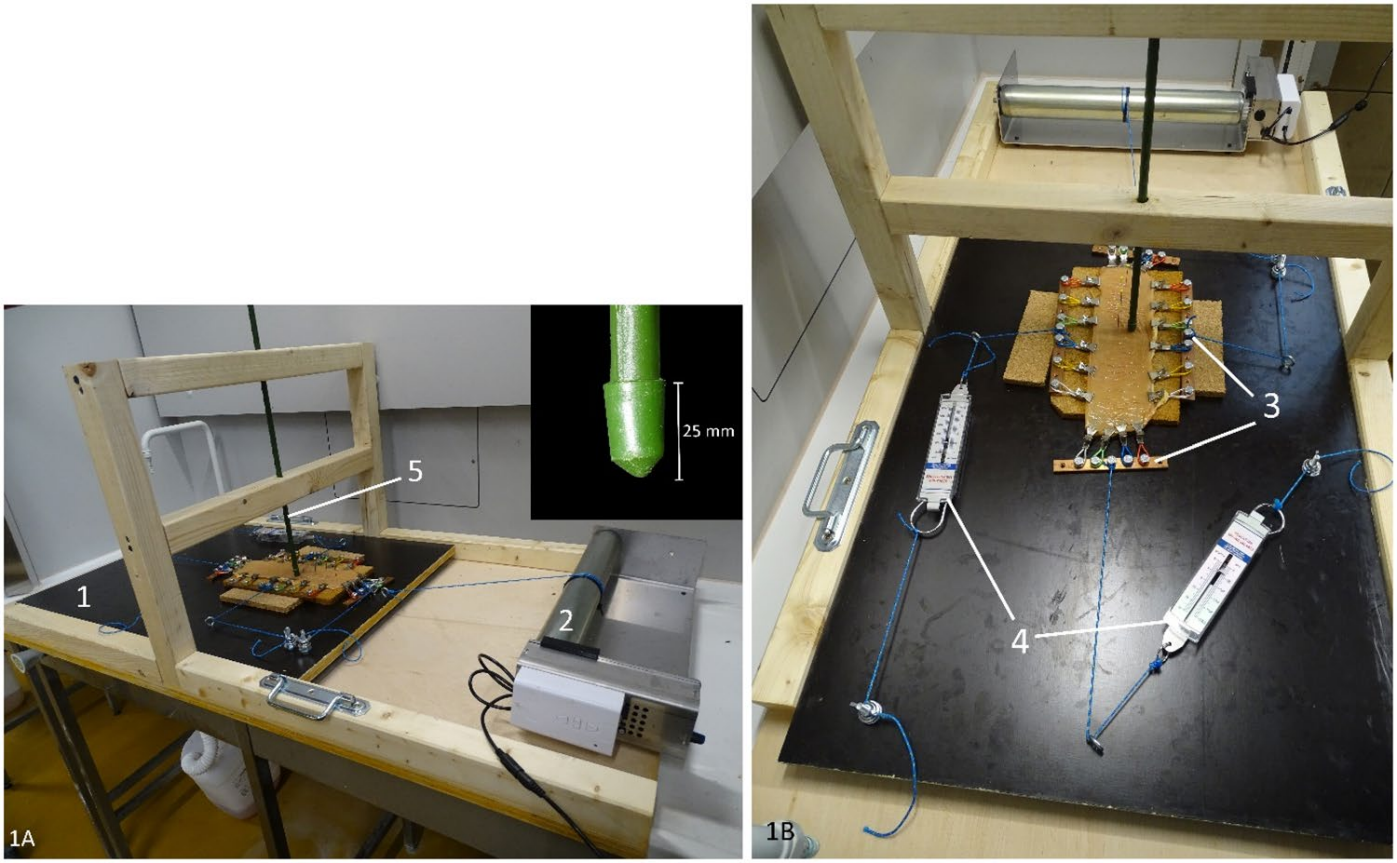
Ex vivo intestinal mucosal abrasion model. The intestine is mounted on the black platform (1) with wheels underneath. It is pulled forward by a pulley winding system (2). The intestinal segment is cautiously stretched in 2 directions with a gripping mechanism (3), the bi-axonal tension is measured with force meters (4). The rod (5) makes contact with the mucosal surface and creates abrasional lesions during forward movement of the platform (close-up of the point of the abrasion rod at the inset).

A device creating mucosal abrasion with a pointed object was developed. In this way, mucosal abrasion can be created in a standardized and reproducible way. The model contains an unidirectional moving platform on which the intestinal segment is mounted and fixed at the four edges using small tooted clamps (20 clamps in total). The platform is pulled forward at a constant speed of 1 cm/sec, using an electronic pulley winding system (Tandwiel.net, Hattem, The Netherlands) (Figure 1A). A gripping mechanism consisting of metal clamps attached to four bars is used to tense up the intestinal specimen at all four sides. A force meter (capacity: 0–20 N, accuracy: 0,1 N, Eisco Labs, US) was used to measure the tension on the intestinal wall in each of 2 dimensions (Figure 1B). The wall tension can be adjusted by manually varying the degree of tightening of the rope attached to the gripping mechanisms. The clamps can rotate at their attachment with the metal bar, allowing elongation of the specimen during extension. To abrase the intestinal mucosa, a fixed rod with a pointed tip was placed perpendicular to its surface (Figure 1A). The weight of the rod can be altered by adding weight in a basket on top of the rod. Abrasional injury of the mucosa was obtained by the one-directional movement of the intestine with the fixed rod allowing contact with the mucosa over a distance of ±15 mm.

#### 2.2.2. Sample collection and preparation

Intestinal samples of non-affected animals were collected at the slaughterhouse of both adult dairy cows (*n* = 4) and adult beef cattle (*n* = 3). From each animal, two segments (+ −50 cm in length), one from the proximal, and one from the distal jejunum, were sampled and immediately transported to the test facility at 4 °C. In cows with HBS (*n* = 3), two samples proximal and two samples distal to the hematoma were taken immediately after euthanasia or death. These three cows are part of the 10 cows with HBS described above.

All intestinal samples were detached from its mesentery, longitudinally opened with scissors, gently cleaned with tap water, and trimmed to 20 cm length. To adjust for variability in contraction of the intestinal wall, intestinal specimens were mounted on the model and biaxially stretched at 4 N, followed by cutting of the specimen at 20 cm in length and 10 cm in width. In this way, more contracted segments are more elongated then less contracted segments, leading to greater length correction, eventually evening out the contraction-effect between samples.

#### 2.2.3. Sample testing

All samples were subjected to the abrasion model using a force of 4, 6 and 8 N combined with a varying weight of the abrasion rod of 33, 67, 98 and 129 g. This results in twelve injury sites at each intestinal sample. When no obvious gross lesion was visible, the scraped area is indicated with a needle. To fit all lesions on the same intestinal sample, the lesions at 8 N are placed parallel to the others. After testing, a picture was taken for gross evaluation. Thereafter, the tensed sample was fixed on a cork plate and suspended in 4% buffered formaldehyde.

#### 2.2.4. Histological grading and ultrastructural examination

**Figure 2.**
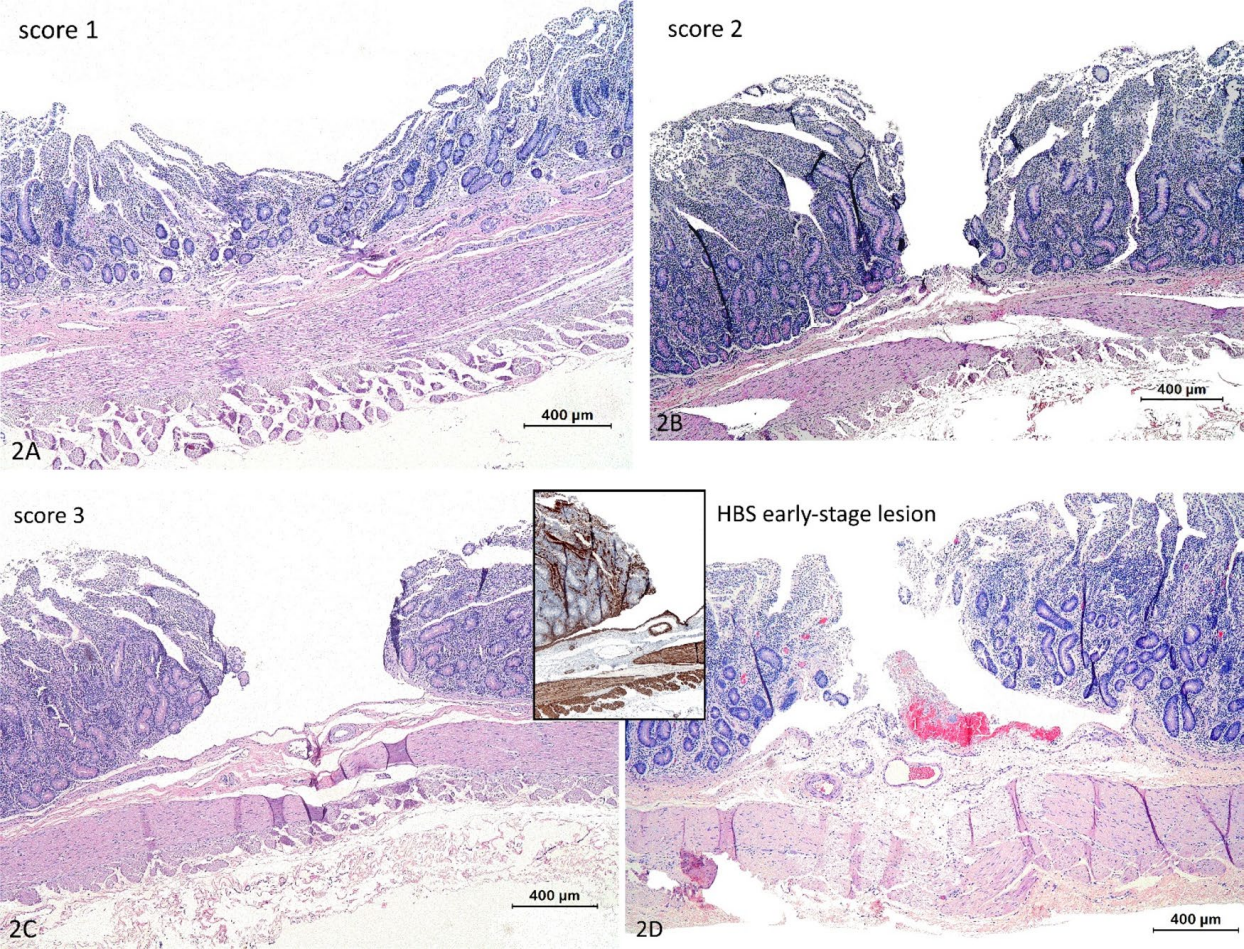
Histological scoring of abrasive lesions created with the ex vivo intestinal mucosal abrasion model. (a) Score 1, depression of the mucosa. Hematoxylin and eosin (HE). (b) Score 2, mucosal cleavage in the propria no dissection of the lamina muscularis mucosae (LMM) (HE). (c) Score 3, mucosal cleavage with dissection of the LMM. (d) Early-stage lesion in a cow with hemorrhagic bowel syndrome. Histologically very similar to score 3, with erosion and dissection of the LMM. Inset: this LMM splitting is even more clear with smooth muscle actin immunohistochemistry. Hemorrhage is evidently only apparent at the early-stage lesion (HE). Magnification = bar.

All lesions were processed as described above and stained with HE. Tissue sections were blindly graded for the following features: (1) mucosal depression (Figure 2a); (2) mucosal cleft in the propria (Figure 2b) (3) mucosal cleft or abrasion with dissection of the LMM at the edges (Figure 2c), similar to early-stage lesions (Figure 2d). If different scores were present within the same lesion, the highest score was recorded. Two lesions with score 3 derived from 2 normal dairy cows, were ultrastructurally examined as described above.

**Figure 3.**
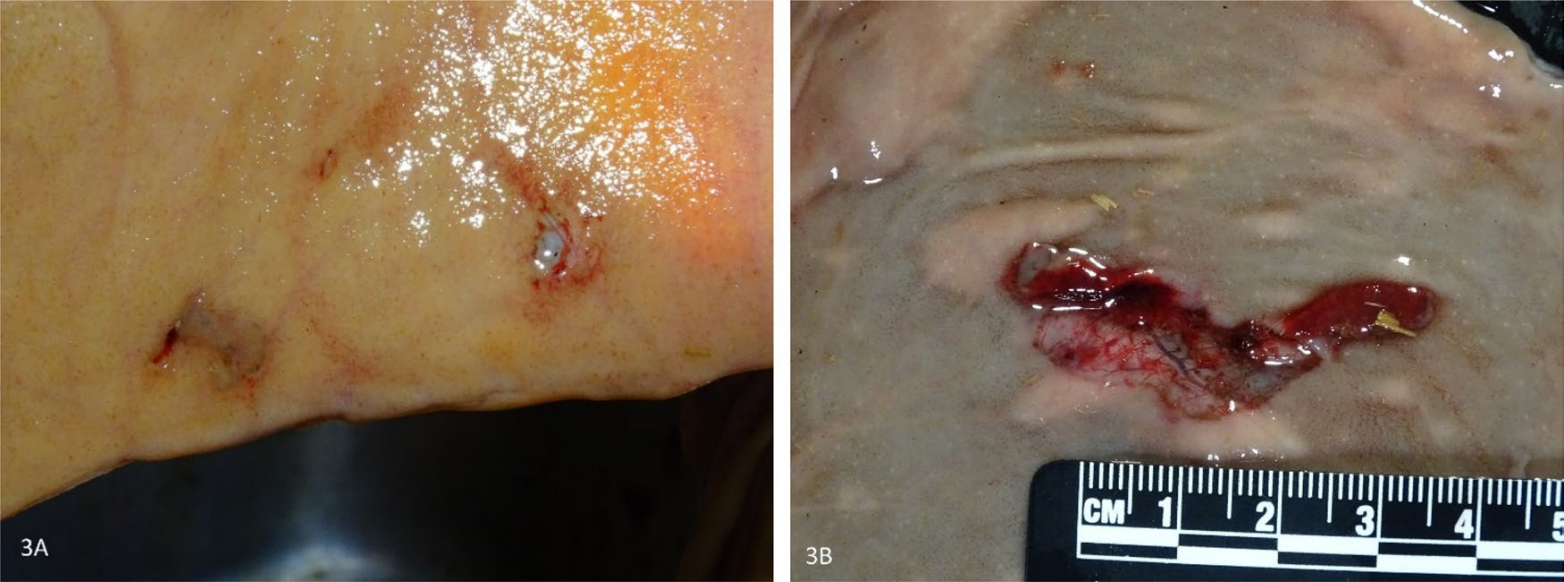
Hemorrhagic Bowel syndrome early-stage lesions, small intestine, bovine. (a) Two clustered erosions with minimal hemorrhage. (b) Laceration-like lesion with a detached mucosal flap and hemorrhage.

## 3. Results

### 3.1. Characterization of early-stage HBS lesions

#### 3.1.1. Gross pathology

**Figure 4.**
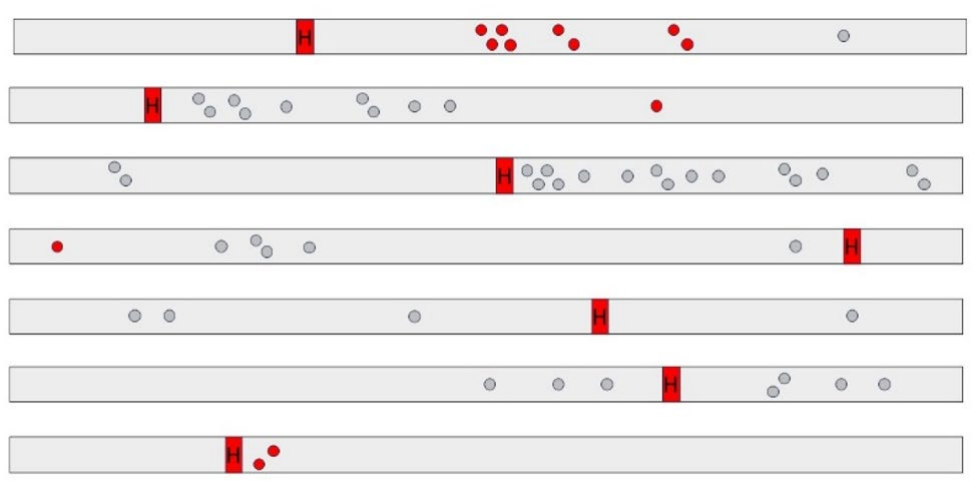
Diagram of gross distribution at the small intestine of early-stage lesions in hemorrhagic bowel syndrome cases (*n* = 7). Red and grey dots resemble early-stage lesions with and without gross hemorrhage, respectively. Lesions tend to occur more commonly proximal to the hematoma. Right to left = proximal to distal. H = hematoma.

In all ten dairy cows with HBS, early-stage lesions presenting as small abrasions with or without hemorrhage were observed. A total of 86 mucosal lesions were counted (mean: 8.6 per cow; range: 1–17 per cow). The diameter varied from 3 to 45mm. Lesions clustered within a few centimeters from each other in 60.5% (52/86) (Figure 3A), or occurred solitary in 39.5% (34/86). Often the mucosa at the edges detached, giving it a laceration-like appearance (Figure 3B). In 39,5% (34/86) of lesions, all deriving from 6 cows, erosions showed hemorrhage. This varied from pinpoint intramucosal hemorrhage, to adhesion of a small blood clot at the eroded surface. In a minority of cases, blood accumulates in the mucosa, giving it the resemblance of a (ruptured) blood blister, with one lesion having the appearance of a small-sized hematoma (diameter 4,5 cm). However, in 60,5% (52/86) of lesions, no gross hemorrhage was apparent. In total, 68 lesions were located proximal to the hematoma, while only 18 were located distally. The hematoma was in all cases located at the jejunum (5/10 distal jejunum, 4/10 mid-jejunum, 1/10 proximal jejunum). The gross distribution of early-stage lesions and the hematoma at the small intestine, was recorded in 7 animals, and are summarized in Figure 4.

#### 3.1.2. Histology and immunohistochemistry

Histologically, 72,1% (62/86) showed erosion until the level of the LMM with detachment of the mucosa at the edges, and splitting at the level of the LMM. The LMM splits with one layer of smooth muscle cells adhered to the detached mucosa, while the other layer of smooth muscle cells kept adhering to the submucosa. This detachment was often extensive, creating a loose mucosal flap. The detached mucosal flap displayed in a small portion of lesions necrosis and hemorrhage. Occasionally, separation of the mucosa from the submucosa occurred just above of the LMM without splitting of this layer. Often the splitting was not precisely in between the 2 layers of smooth muscle, with cells of the upper or lower layer lifting off with the other side. In 12,8% (11/86), expansion of the LMM with hemorrhage, resembling a hematoma was apparent. In 14,0% (12/86) and 9,3% (8/86) of lesions, the erosion did not split the LMM or reached the submucosa, respectively. In 36% (31/86), no fibrin or extravasation of red blood cells was present.

At immunohistochemical examination and reticulin staining, no difference compared to the controls was present. At PAS staining, in two animals a mild PAS-positive granular material in smooth muscle cells was apparent at the tunica muscularis and LMM of both early-stage lesion and normal intestine.

#### 3.1.3. Electron microscopy

**Figure 5.**
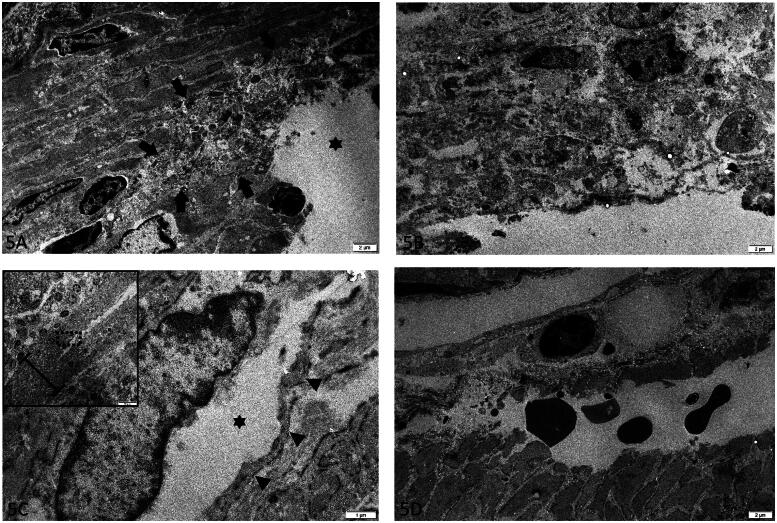
Lamina muscularis mucosa (LMM) at different early-stage lesions (a-c) and hematoma (d) in hemorrhagic bowel syndrome (HBS) cases. **(a-b)** Focally (indicated with arrows) at the ulcerated surface (asterisk) (a) to diffuse (b), degeneration of smooth muscle cells are present at the LMM. There is severe fragmentation and swelling of organelles, rarefaction and chromatin condensation. **(c)** De splitting of the LMM occurs through ruptured smooth muscle cells. The cell membrane of the splitted cell is indicated with arrow heads. The dissection (asterisk) cuts through the cytoplasm, adjacent to the nucleus. Inset: lower magnification overview of the splitting area of the LMM, with the area shown in ‘5C’ indicated with a rectangle. The LMM is indicated with an accolade **(d)** Hematoma at the start of the dissection. The cleavage of the LMM is confined to the upper portion of the inner layer of smoot muscle. Extravasated red blood cells are in between the splitted layers. TEM. Magnification = bar.

In three out of four early-stage lesions derived from three cows with HBS, degeneration of smooth muscle cells was present at area’s where splitting of LMM was present. Degeneration was restricted to cells at the eroded surface of the LMM, and was focal in 1/3 (Figure 5A), and diffuse in 2/3 (Figure 5B). Degenerative changes were characterized by swelling and fragmentation of organelles, loss of myofilaments, rarefaction and condensation of chromatin. At the hematoma however, degenerative changes were absent or minimal. Furthermore, the splitting within the LMM was cutting through both the intercellular matrix of smooth muscle cells, as through the cell bodies (Figure 5C). This was the case for both early-stage lesions and hematoma. In addition, at the hematoma, the splitting was confined within the upper layer (of the two layers) of smooth muscle cells of the LMM (Figure 5D). Blood vessels within the submucosa and propria were considered within normal limits.

#### 3.1.4. Bacteriology

Non-selective culture demonstrated abundant growth of *Escherichia coli* at all but one lesion (14/15). Fewer colonies of a multitude of other bacterial species were isolated, with *Clostridium perfringens* being the only one recurrently present in all 6 animals. It was isolated in 10/15 lesions. Other isolated bacteria were *Escherichia fergusoni*, *Bacteriodes spp*., *Fusobacterium varium* and *Paeniclostridium sordelli*. The latter two were only present at all lesions in one animal. In 4 lesions from 1 animal, the aerobic plates were excluded because of overgrowth by *Proteus*, preventing purification. With CHCP anaerobic culture, *C. perfringens* was isolated in 14/15 lesions, with growth covering less than 33% of the streaked area in 9/14 lesions, and more than 33% in 5/14 lesions. An overview of the results is given at Table 1.

**Table 1.** Overview of bacterial culture of 15 early-stage lesions, in 6 cows with hemorrhagic bowel syndrome (HBS).

Case *N*°	1	2				3					4		5	6	
**Lesion *N*°**	1	2	3	4	5	6	7	8	9	10	11	12	13	14	15
*Escherichia coli*	2	2		3	3	2	4	7	5	3	5	2	2	9	8
*Escherchia fergusoni*	1					1	1				1				
*Proteus mirabilis*	1	1*	1*	1*	1*										
*Clostridium perfringens*	1		1	1			1	1			5	2	2	2	2
*Bacteroides spp.*		4	3	1	2				1						
*Basfia succiniproducens*						2				1		1			
*Fusobacterium varium*						3	3	3	2	1		1			
*Paeniclostridium sordelli*						1	2	1	2	1					
*Paraclostridium bifermentans*											1	2		2	1
*Bacillus pumilus*							1			1					
CHCP	+	++	++	++	++	+	+	+	+	+	++	+	–	+	+

The number of identified bacterial species isolated with non-selective bacterial culture are given. Only bacteria isolated more than once are listed. The amount of growth of C. perfringens at selective culture (CHCP) was estimated (0, no growth; +, <33% of streaked area; ++, >33% of streaked area). 1*, aerobic plates were overgrown by Proteus.

### 3.2. Ex vivo intestinal mucosal abrasion model

**Figure 6.**
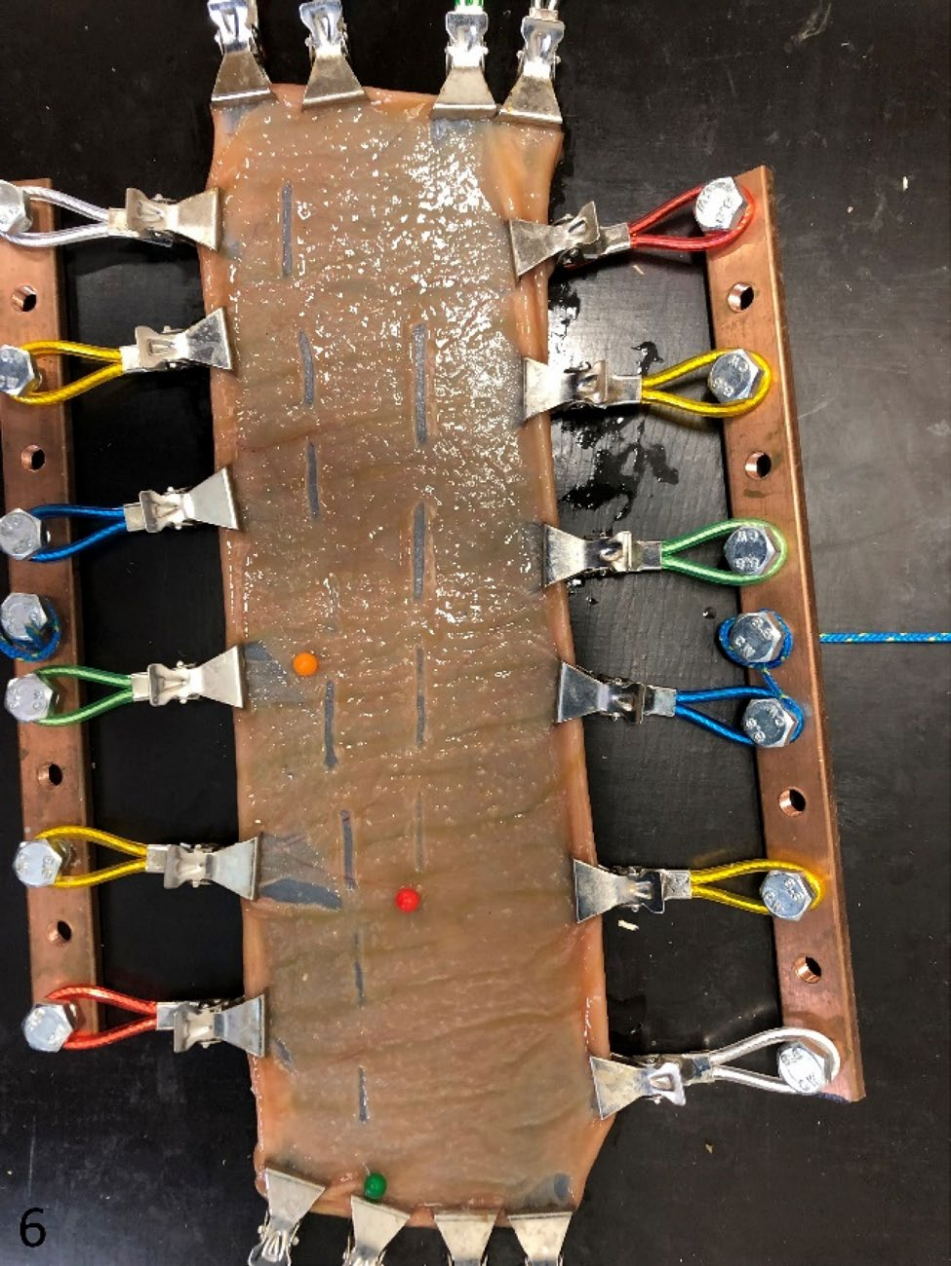
Mounted intestinal segment at the end of the experiment. Twelve injurie sites are evident. The intestine is tensed up by the gripping mechanism. The needle head color refers to the tension used for the following 4 lesions (green, 4 N; orange, 6 N; red, 8 N).

Grossly, the abrasion lesions varied from no gross change to linear mucosal cuts over the entire length of injury site (Figure 6). Ultrastructurally, abrasive lesions (*n* = 2), showed no degeneration or other changes. The splitting within the LMM was cutting through both the intercellular matrix of smooth muscle cells as through the cell bodies, as was the case for early-stage lesions and hematoma (see above).

**Figure 7.**
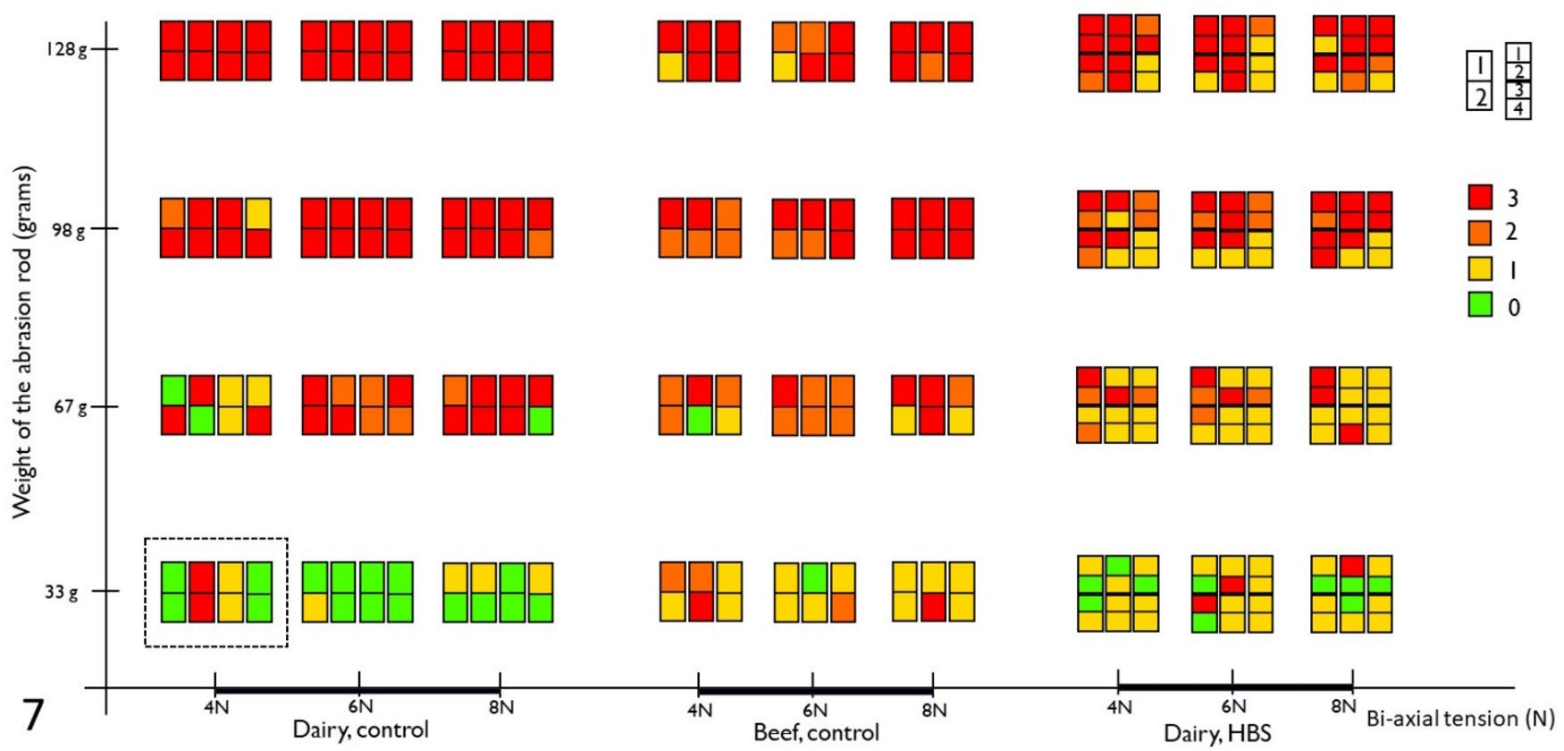
Summary of histological lesions scores of intestinal segments after testing with the *ex vivo* intestinal mucosal abrasion model. Results of 7 control animals (4 dairy and 3 beef cattle), and 3 cases of hemorrhagic bowel syndrome (HBS), are displayed. Each colored rectangle depicts results of one animal, for one combination of bi-axial tension and rod pressure. The dotted rectangle, surrounds results from 4 animals, 8 segments for the combination of 33 g and 4 N. The colors indicates the histological lesion score. Numbering of segments, as shown on the figure, are from proximal to distal.

The histologic scoring results are summarized in Figure 7. In both normal dairy cow (*n* = 4), normal beef cattle (*n* = 3) and dairy cows with HBS (*n* = 3), mucosal abrasions with prominent dissection of the LMM could be created, similar to early-stage lesions (Figure 2D). With increasing weight of the abrasion rod, lesion scores tend to be higher in all animals, resulting in predominantly abrasion with dissection of LMM (score 3) at 128 g rod weight. Also, with increasing wall tension, higher lesion scores can be observed at probe weight 67 g and 98 g in normal animals, and at 98 g in intestine proximal to the hematoma in HBS cases. In addition, in dairy controls compared to beef controls, a higher proportion of lesions shows score 3 at 67 g and 98 g in all 3 tensions. However, lesion scores were similar for proximal intestine in HBS dairy cows in comparison with normal beef cattle. At 33 g probe abrasion weight, a higher portion of lesions in normal dairy cows had score 0 compared to the others. Furthermore, intestine proximal to the hematoma, showed more severe lesions compared to intestine distal to the hematoma in HBS cases.

## 4. Discussion

This study was conducted to better understand the pathogenesis of HBS. Dissection of the LMM is a unique microscopic feature of both the hematoma and early-stage lesions in HBS. They are most likely manifestations of the same disease process, with early-stage lesions presumed to be the initial lesion from which the hematoma develops. Detachment and tearing in between the two smooth muscle layers at this level, appears to be the most early event in the disease pathogenesis. Since, animals surviving HBS, tend to have a re-occurrence of the disease and certain breeds are predisposed (Peek et al. 2009; Braun et al. 2010), it should be considered if any structural alteration at the LMM is present, causing some kind of fragility at this level. The first aim of this study was to investigate this by evaluating the morphology of the LMM at the microscopic and ultrastructural level. Also, gross pathology and bacterial culture results were reported.

Early-stage lesions did occur both proximal and distal to the hematoma, they were however more common proximal and tend to increase in number approximating the hematoma from the proximal side. This is different to what was previously described, as it was reported to only be present proximal to the hematoma (De Jonge et al. 2023). In three animals, the lesion density, seems to increase when approximating the hematoma from proximal to distal. This might indicate some of these lesions developed after the hematoma already was present (see below). Furthermore, gross and histological appearance of these early-stage lesions were in line to what was previously described by De Jonge et al. (2023). Histologically, the detached mucosal flap of a few lesions showed necrosis and hemorrhage. This was believed to be secondary to ischemia and exposure to intestinal contents after it already detached.

Electron microscopical examination shows degenerative changes at the smooth muscle cells of the LMM in 3/4 early-stage lesions, but not at the hematoma and control samples. This indicates cellular injury of smooth muscle cells at the early-stage lesions. It is however unclear if this is a primary or secondary lesion. In the latter case, exposure of smooth muscle cells to the harmful ingesta or cell injury caused by the tearing apart of the LMM could be the cause. No to minimal degeneration was present at the LMM of the hematoma. This might be explained by the rapid dissection and progression of the hemorrhage, leaving not enough time at the edge (where the sample was taken) to develop degeneration. Another interesting finding was the fact that the dissection in both early-stage lesions, hematoma and trauma induced controls, cut through the intercellular matrix and through smooth muscle cells. If the underlying cause for HBS would be an alteration of the intercellular matrix or cellular adhesion to intercellular matrix, it could be expected that the splitting would be consistently located there and not also through smooth muscle cells. As is the case for bullous pemphigoid for example, where anti-hemidesmosome antibodies affect the detachment of epidermal keratinocytes on their basal membranes, with eventually detachment of cells (Giannotti et al. 1975).

Nonselective bacterial culture of mucosal swabs of early-stage lesions, showed a polybacterial culture dominated by *E. coli* at the early-stage lesions. *Clostridium perfringens* type A (Beta2+), which was reported to be associated with HBS in several previous studies (Kirkpatrick et al. 2001; Dennison et al. 2002; Ceci et al. 2006), was recovered in the majority of lesions with both nonselective and selective culture. *Clostridium perfringens* can be isolated from normal intestine as well as intestine containing hemorrhagic contents independent of the underlying condition (Adaska et al. 2014). As reported before, mainly based on histological findings, it is unlikely for *C. perfringens* to be the cause of HBS since microscopic pathology is very different to well-known clostridial enteropathies (Adaska et al. 2014; De Jonge et al. 2023). Drawing conclusions from these culture results is only possible to a limited extent, since no controls were used. It is however debatable what a good control would be since stasis of ingesta and blood altering the microbial environment, was most likely present for more than 12 h before sampling. Although, if a bacterial infection would be the underlying cause of HBS, high numbers and pure or abundant culture of a single bacterium would be expected (Songer 1996), which is not the case here. Also, histological appearance is not suggestive of a primary bacterial infection at the early-stage lesions (De Jonge et al. 2023).

A second aim of this study was to investigate if trauma to the mucosa plays a role in the pathogenesis of HBS. This hypothesis is based on the fact that early-stage lesions show no underlying histological changes which might explain their occurrence and they sometimes have a laceration-like appearance (De Jonge et al. 2023). And, if this would be the case, are there any indications for an underlying weakness or fragility of the LMM in cows with this disease? This was explored by using an *ex vivo* model.

The most important finding of this study, is that mucosal abrasion was able to create lesions histologically very similar to early-stage lesions. Indeed, both in normal dairy cows and beef cattle as well as in HBS dairy cows, lesions showing erosion of the mucosa with splitting of the LMM at the edges could be created. This closely resembles early-stage lesions as described by De Jonge et al. (2023). Splitting of the LMM is a highly unique feature of HBS, to the author’s knowledge, similar splitting of the muscularis mucosae is not described in any intestinal pathology. This splitting of the LMM is also consistently present at the hematoma, as this is a dissecting hemorrhage of this intestinal layer. Because of consistent splitting at this level, it seems likely this layer is a weak spot when abrasive injury is occurring to the mucosa, causing it to rupture and split if enough mechanical stress is present. Taking the histological findings and gross appearance of early-stage lesions into account combined with these results, we find it reasonable to suggest that mucosal trauma might play a role in the pathogenesis of these early-stage lesions and so HBS.

HBS seems to recurrently target individual animals. It is known that cows surviving HBS, are often experiencing another HBS episode weeks to months later (Peek et al. 2009; Braun et al. 2010). Also, Brown-Swiss and derived breeds seem to be predisposed to this disease (Peek et al. 2009; Braun et al. 2010). This could indicate that a factor linked to the animal itself plays a role in the development of HBS, rather than only external factors. Therefore, it was hypothesized by the authors, that some kind of fragility or weakness of the LMM smooth muscle tissue, could predispose to detaching of smooth muscle, secondary erosion and hemorrhage, followed by further dissection of LMM during the progression of the hematoma. So, if some kind of fragility was present, a higher lesion score at the different rod weights and wall tensions compared to controls, could be expected in animals with HBS. However, results in the current study do not show higher lesions scores in HBS cows. Nevertheless, more severe lesions were apparent in normal dairy cows compared to normal beef cattle. This could be an interesting finding, since HBS typically targets dairy cattle. It needs to be investigated with higher numbers of animals and after further validation of this model, if this is a real difference or not. Also, as mentioned above, no morphological alterations at the LMM could be detected with histochemical or immunohistochemical examination in cows with HBS. In addition, even if some kind of fragility of the LMM would play a role in development of HBS, this does not exclude concurring mucosal trauma, caused by (physiological) mechanical forces created by peristalsis and ingesta passage, initiates early-stage lesion formation.

Rod pressure and stretching of the intestinal wall, seem to be determining factors in whether abrasion leads to dissection of the LMM using this *ex vivo* model. Results show a tendency for higher (more severe) lesions scores, when a higher abrasion pressure (rod weight) or intestinal tension was applied. Stretching of the mucosa reduces mucosal folding and makes the mucosa thinner, and more easy to be breached by the abrasion rod during the experiment. Also, in HBS cases, the intestine proximal to the hematoma showed on average a higher lesion score at the different abrasion pressures and wall tensions than the distal intestine. This might possibly explain why these HBS early-stage lesions are more commonly occurring proximal to the hematoma, and in higher density approximating to the hematoma. The hematoma obstructs the intestine causing ingesta accumulation proximal to it, which creates distention and stretching of the intestinal wall. This might predispose to development of trauma-induced mucosal lesions. This would however imply that these lesions, or at least a proportion, came to existence after the hematoma was already formed. Stretching of the intestinal wall might also be a predisposing factor in the initial stage of the disease, for example by dilation of the intestine with feed stuff or gas.

If mucosal abrasion is the underlying cause of HBS early-stage lesions, the injuring material would be derived from the intestinal lumen. It remains however an unanswered question what the precise nature of this material would be. In the normal ruminant forestomach, only omasal passage of sedimented small digesta food particles occurs, while larger particles stay in the rumen (Beauchemin 2018). In modern high-producing dairy cows, digesta particle size without increased resistance for ruminal escape (critical size threshold), is 1,18 mm (Maulfair et al. 2011). However, in cows with HBS, often a large amount of undigested corn silage was present in the abomasum, often combined with a ruminoreticulum distended with impacted content (De Jonge et al. 2023). It is unclear whether this was a secondary phenomenon due to intestinal obstruction created by HBS, or a primary event. HBS typically affects lactating dairy cattle, animals with high dry matter uptake (Elhanafy et al. 2013). Also, management practices aimed for high milk yield are known risk factors for HBS (Berghaus et al. 2005). In dairy cows with high feed uptake, particle size of digesta leaving the rumen increases (Shaver et al. 1988). Possibly, high feed uptake could result in passage of undigested feed stuff, which could cause micro-abrasion to the small intestinal mucosa during peristalsis. Further research is necessary to explore this hypothesis. Nevertheless, HBS seems to be a sporadic and more individual animal problem, so this theory is not completely explanatory.

This study is subject to several limitations. First, two intestinal samples from the same animal tested with the *ex vivo* model, show often unidentical lesion scores, although they are expected to be the same. This most likely indicates that, although sample preparation already tries to correct for it, intestinal contraction still influences experiment outcome to a certain degree. Further validation of this model is necessary. Secondly, only a limited number of samples were evaluated with electron microscopy. Also, the number of animals tested with the *ex vivo* model was limited, allowing no statistical analysis. Third, no controls were used with the bacterial examination. Fourth, for several early-stage lesions, it is not entirely clear, even at the histological level, if they were in fact lesions or artefact (for example because of postmortem manipulation of the intestines). This was especially so when lesions did not demonstrate hemorrhage or fibrin deposition.

Future research should test this mucosal abrasion theory in an *in vivo* model. This could elucidate if lesions caused by mucosal injury could develop into a hematoma through dissection of the LMM. Also, further studies should focus on why individual animals can be predisposed to HBS. This could be done by studying gastro-intestinal physiology and eating behavior of animals which survived an episode of HBS, for example with sensors or ultrasonography.

In conclusion, this study shows that lesions very similar to early-stage lesions, can be created with mucosal abrasion. This might indicate that mucosal trauma (e.g. abrasion caused by mechanical injury coming from the intestinal lumen), might play a role in the pathogenesis of hemorrhagic bowel syndrome. A precise cause for possible mucosal abrasion remains elusive. The consistent splitting of LMM at early-stage lesions and experimentally induced abrasive lesions, is most likely because this smooth muscle layer is the weakest spot, prone to split when enough mechanical stress is applied. There is no indication for a morphological alteration at the LMM which could explain some kind of weakness or fragility. However, degenerative changes of the LMM at the ultrastructural level of early-stage lesions are present, it is however unclear if they have a primary or secondary nature. The findings of this study may aid in further hypothesis generation to unravel the pathogenesis of HBS.

## Data Availability

On request.
